# The importance of N6-methyladenosine modification in tumor immunity and immunotherapy

**DOI:** 10.1186/s40164-022-00281-2

**Published:** 2022-05-19

**Authors:** Ze Zhang, Furong Liu, Wei Chen, Zhibin Liao, Wanguang Zhang, Bixiang Zhang, Huifang Liang, Liang Chu, Zhanguo Zhang

**Affiliations:** 1 Hepatic Surgery Center, Tongji Hospital, Tongji Medical College, Hubei Province for the Clinical Medicine Research Center of Hepatic Surgery, Huazhong University of Science and Technology, 1095 Jiefang Avenue, Hubei 430030 Wuhan, China; 2 Hubei Key Laboratory of Hepato-Pancreato-Biliary Diseases, 430030 Wuhan, Hubei China

**Keywords:** N6-methyladenosine, Tumor immunity, Immune escape, Immunotherapy

## Abstract

As the most common and abundant RNA modification in eukaryotic cells, N6-methyladenosine (m6A) modification plays an important role in different stages of tumor. m6A can participate in the regulation of tumor immune escape, so as to enhance the monitoring of tumor by the immune system and reduce tumorgenesis. m6A can also affect the tumor progression by regulating the immune cell responses to tumor in tumor microenvironment. In addition, immunotherapy has become the most popular method for the treatment of cancer, in which targets such as immune checkpoints are also closely associated with m6A. This review discusses the roles of N6-methyladenosine modification in tumor immune regulation, their regulatory mechanism, and the prospect of immunotherapy.

**Graphical Abstract:**

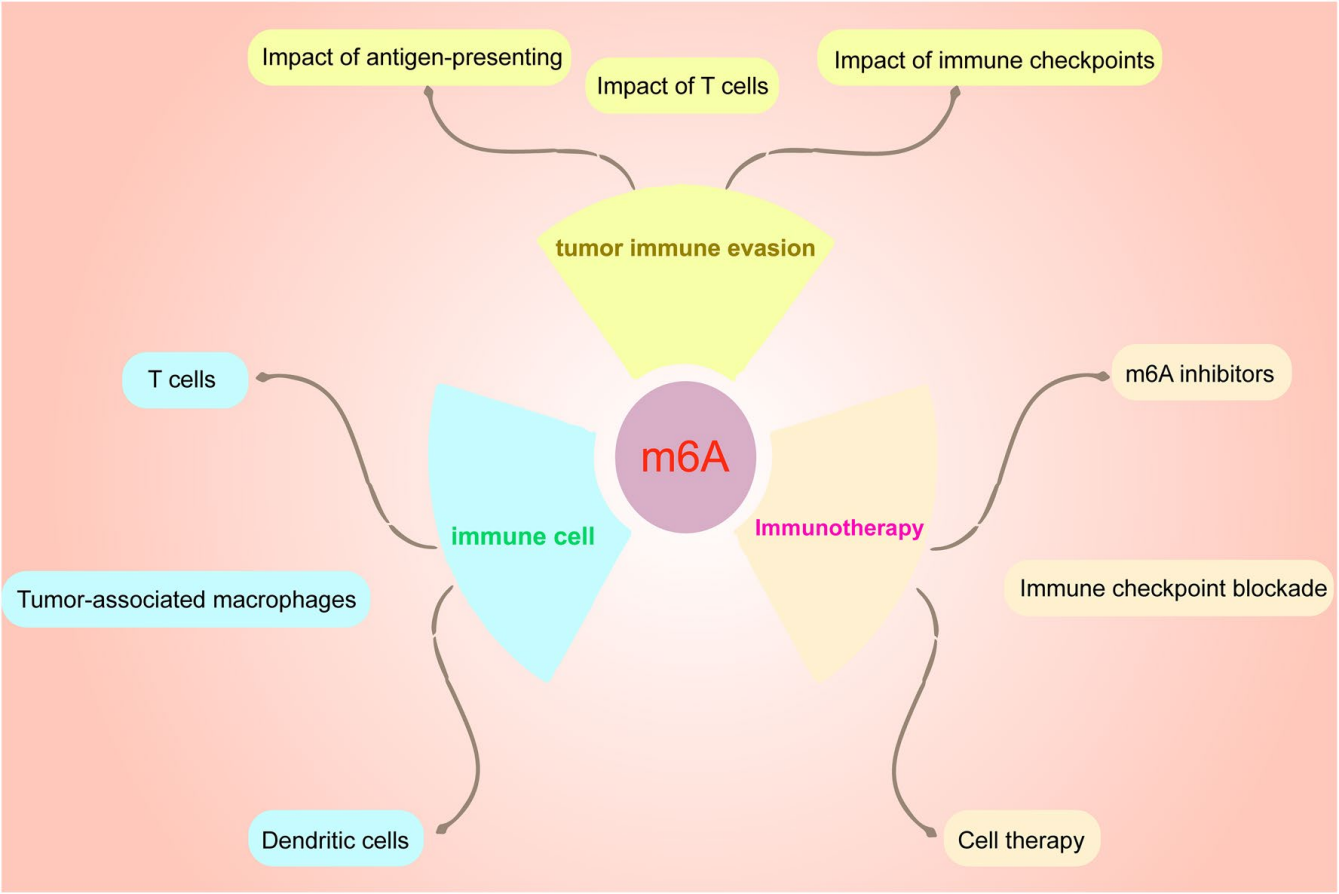

## Introduction

According to the data, more than 160 different types of RNA chemical modifications have been identified in all living organisms so far [1], including N6-methyladenosine (m6A), N1-methyladenosine (m1A), 5-methylcytosine (m5C), N7-methylguanosine (m7G), RNA cap methylations and so on. Among these modifications, N6-methyladenosine (m6A) is the most common and abundant RNA modification in eukaryotic cells [2, 3]. As a form of epigenetic regulation, m6A is a dynamic and reversible process, which is modulated by various enzymes in the mammalian cells [4]. N6-methyladenosine is enriched near stop codon and 3′ untranslated terminal region (UTR), and translated near 5′ UTR in a cap-independent manner as a means of fulfilling its function [5–7]. The function and mechanism of m6A modification have been widely studied in recent years, which is involved in many aspects of RNA metabolism, including pre-mRNA splicing, nuclear export, 3′-end processing, translation regulation, mRNA decay and noncoding RNA (ncRNA) processing [8–10]. A large number of studies have shown that m6A plays an important role in almost all biological processes, especially in tumorigenesis [11]. Recent studies have revealed that m6A can affect tumor progression through the regulation of immunity, which has become a research hotspot. In this review, we mainly discuss the abundant regulatory role of m6A in tumor immunity and escape mechanism, as well as tumor immunotherapy.

## m6A writers, erasers and readers

m6A is catalyzed by RNA methyltransferase complex, which is composed of several components and called “writers” [12]. Methyltransferase-like 3 (METTL3), the first characteristic component of RNA methyltransferase complex, is a S-adenosylmethionine (SAM)-binding subunit, which mainly acts as the catalytic core [13, 14]. Methyltransferase-like 14 (METTL14) is another active component of m6A methyltransferase complex, which mainly functions as a supporting structure [14]. METTL3 and METTL14 coexist in the nuclear spots at the ratio of 1:1 and form a stable complex [15]. WTAP (Wilms tumor-associated protein) is the third core component of m6A methyltransferase complex. The main function of WTAP is to act as a junction protein for the interaction between METTL3 and METTL14, but without catalytic activity [16]. WTAP can also interact with many other proteins and long non-coding RNAs(lncRNAs), suggesting that WTAP may recruit other factors to the methyltransferase complex [4]. METTL16 is an independent methyltransferase, which can catalyze m6A of mRNAs, lncRNAs and U6 small nuclear RNA [17]. In addition, KIAA142929 [18], RNA binding motif protein 15 (RBM15) and its analogue RBM15B30 [19] are all part of the RNA methyltransferase complex, which jointly catalyze the methylation of RNA.

Fat mass and obesity-associated protein (FTO) was the first m6A demethylase to be discovered [20], and then α-ketoglutarate-dependent dioxygenase homolog 5 (ALKBH5), was soon excavated [21]. Both FTO and ALKBH5 belong to the family of α-ketoglutarate-dependent dioxygenases, which are ferrous iron and α-ketoglutarate (αKG) cofactors-dependent demethylases [21]. They are mainly located in the nucleus where m6A demethylation occurs. Although the contents of FTO and ALKBH5 are different in various tissues [22], they both regulate the level of m6A in cells by reversing m6A modification. ALKBH3 is also an m6A demethylase, but it is more likely to modify tRNA than mRNA or rRNA [23]. The revealing of m6A demethylase indicates that m6A methylation is a reversible process, which enlightens the exploration of function and mechanism in m6A modification.

**Fig. 1:**
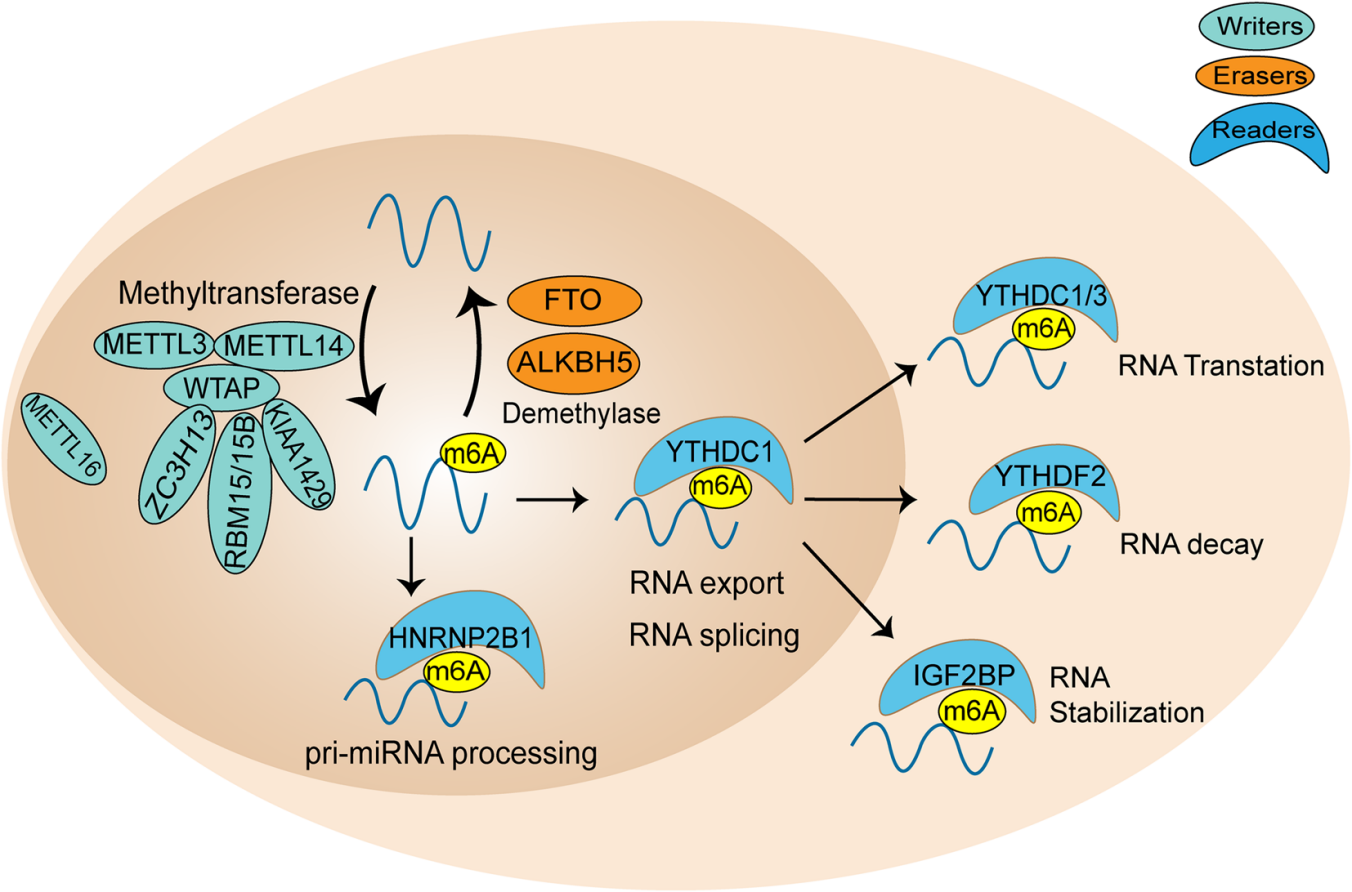
m6A writers, erasers and readers. “Writers” can catalyze m6a modification, while “erasers” can reverse this process. “Readers” recognize the bases of m6A methylation, accelerate the nucleation rate of mRNA, and participate in RNA translation, RNA decay and RNA stabilization

The secondary or tertiary structure of m6A-modified RNA will be changed, while “readers” can decode the m6A RNA methylation information in the cell [24, 25]. The “Readers” consist of YTH domain-containing proteins (YTHDF1/2/3 and YTHDC1/2), heterogeneous nuclear ribonucleoproteins (including hnRNPC, hnRNPG and hnRNPA2B1) and insulin-like growth factor 2 mRNA-binding proteins (IGF2BP) [26, 27]. The function of YTHDF1 is to interact with translation initiation factors to facilitate the translation efficiency of RNA [28]. In contrast, YTHDF2 accelerated the decline of m6A modified transcripts by directly recruiting the CCR4-NOT complex [29, 30]. YTHDF3 synergistically promotes protein synthesis and antagonizes YTHDF2-mediated mRNA decline [31–33]. As the only nuclear m6A “reader” protein, YTHDC1, also called YT521-B, has many functions, including recruiting some splicing factors to regulate the splicing of mRNA [34], promoting the output of mature mRNA [35] and the decay of specific transcripts [36]. While YTHDC2 can regulate mRNA stabilization and translation [37–39]. The functional core of IGF2BP protein is the KH3-4 domain, which can recognize m6A modification to enhance the stability and translation of its target mRNA [40]. HnRNPA2B1 regulates the alternative splicing of exons and interacts with Dgcr8, a pri-miRNA microprocessor complex module, to regulate the nuclear processing of pri-miRNA (Fig. 1) [41].

## m6A in tumor immunity

### Regulation of m6A on immune cells

#### Tumor-associated macrophages (TAMs)

**Fig. 2:**
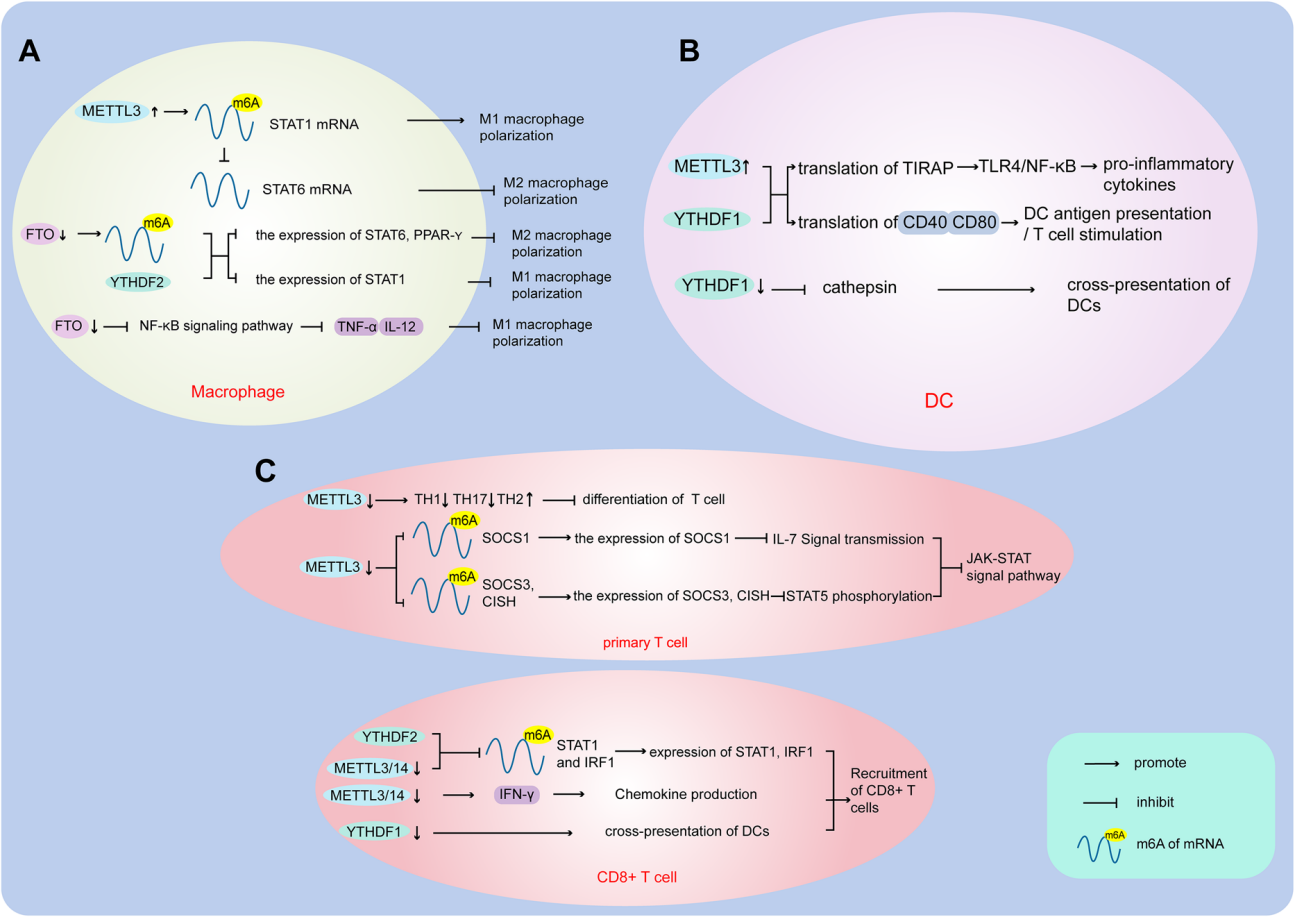
Regulation of m6A on immune cells. **A** The modification of m6A can regulate the polarization of macrophages. **B** The modification of m6A can regulate antigen presentation of DCs and T cell stimulation. **C** The modification of m6A can regulate the homeostasis of T cells

Tumor-associated macrophages (TAMs) are important regulators of tumorigenesis, which can exist in tissues or originate from peripheral hosts such as bone marrow (BM) and spleen [42]. Although macrophages are generally considered to be key effector cells in the process of immune defense [43], a large number of studies have proved that TAMs have a clear role in supporting tumor progression in many aspects. The two well-characterized extreme phenotypes of macrophages are M1 macrophages, and M2 macrophages [44, 45]. M1 macrophages are involved in pathogen clearance during infection by activating the nicotinamide adenine dinucleotide phosphate (NADPH) oxidase system and subsequently generating reactive oxygen species (ROS) [46, 47]. M2 macrophages have strong phagocytosis ability, promote tissue repair, scavenge debris and apoptotic cells, and possess pro-angiogenic and pro-fibrotic properties [43, 45, 48]. In general, M1 macrophages mediate tissue damage induced by reactive oxygen species, and are helpful in antimicrobial and antitumor activities [43, 49]; M2 macrophages are involved in Th2 cell involved immune response and inflammatory suppression, and promote tissue remodeling, angiogenesis, tumor formation and progression [46, 50]. Studies have shown that m6A can regulate the polarization of macrophages by different mechanisms (Fig. 2A). Liu et al. found that METTL3 was specifically upregulated following polarization of mouse M1 macrophage [51]. Signal transducer and activator of transcription 1 (STAT1), is a major transcription factor that controls the polarization of M1 macrophage [52]. As a methyltransferase, METTL3 enhances the stability of STAT1 mRNA by specifically increasing the m6A modification of STAT1 mRNA in murine bone marrow-derived macrophages, and upregulates the expression of STAT1 mRNA to facilitates M1 macrophage polarization. On the other hand, the activation of STAT1 also inhibited the transcription activity of STAT6 which is the key transcription factor of M2 macrophage polarization mediated by IL-4. As a result, up-regulation of METTL3 facilitates M1 macrophage polarization and meanwhile inhibits M2 macrophage polarization [51]. Hence, METTL3 is involved in regulating the balance polarization of M1 and M2 macrophages. Besides, as a demethylase, FTO can also regulate M1 and M2 macrophage activation [53]. After FTO was knocked down in mouse macrophages, STAT1 expression was down-regulated in M1-polarized macrophages, while STAT6 and PPAR-γ expression was decreased in M2-polarized macrophages. FTO knockdown accelerates the mRNA decay of STAT1 and PPAR-γ in macrophage activation [44, 54], and this process is also related to the involvement of YTHDF2, which has an effect on the stability of the mRNA of STAT1 and PPAR-γ. Moreover, FTO knockdown inhibits the activity of NF-κB signaling pathway, a main activation pathway for M1, and the activation of which promotes the expression of inflammatory factors TNF-α and IL-12 [55]. Last but not least, FTO knockdown can inhibit the phosphorylation of NF-κB signaling pathway in the activation of M1 macrophages, resulting in the suppression of the activation process of M1 macrophages [53]. Similarly, RBM4 overexpression can decrease m6A modified STAT1 mRNA to inhibit glycolysis in RAW264.7 macrophages by interacting with YTHDF2, thereby suppressing IFN-γ-induced M1 macrophage polarization [56]. In brief, FTO knockdown and RBM4 overexpression both inhibited the polarization of M1 macrophages, which is not advantageous for immune control of tumors.

On the other hand, the study found that deletion of METTL14 in myeloid cells exasperates the macrophage response to acute bacterial infection in mice [57]. Deletion of METTL14 in myeloid cells decreases methylation of SOCS1 mRNA, which maintains negative feedback control of macrophages. This leads to excessive activation of TLR4/NF-κB signaling pathway and release of excessive pro-inflammatory factors to enhance the inflammatory response [57]. This shows that m6A can be involved in the regulation of macrophage activation to exert anti-inflammatory effects, again demonstrating the important role of m6A on macrophage polarization.

#### Dendritic cells (DCs)

DC cells are a kind of antigen presenting cells, which can absorb, process and transmit antigen information in the body, and induce T and B cells to produce immune responses [58, 59]. In a study, Wang et al. found that METTL3 promotes dendritic cell activation by utilizing DC maturation and a differentiation model, including bone marrow-derived immature dendritic cell, LPS-stimulated BMDC (mature DC), and regulatory DC [60]. METTL3 promotes DC maturation in a m6A dependent manner, during which m6A modification level is increased. When METTL3 in DC cells is knocked out, TLR4/ NF-κB signaling pathway is defective, and IL-6 and IL-12 mRNA levels are decreased. This phenomenon suggests that METTL3 may promote the expression of NF-κB signaling pathway during DC maturation and activation through TLR4/NF-κB signaling pathway. TIRAP, an adaptor in the TLR4/NF-κB signaling pathway [61], was subsequently found to be involved in the regulation of this process. In general, METTL3 can promote TIRAP, CD80, CD40 mRNA translation in vivo and in vitro, which is positively correlated with YTHDF1. The up-regulation of TIRAP expression facilitated the enhancement of TLR4/ NF-κB signaling pathway and the secretion of pro-inflammatory cytokines, while the up-regulation of CD40 and CD80 expression led to the increase of DC antigen presentation and T cell stimulation [60]. Therefore, in vitro and in vivo, METTL3 promotes T cell proliferation through its m6A catalytic activity, which is required for DC function.

In addition m6A modification of mRNA is closely related to the regulation of persistent neoantigen-specific immunity of DC cells through the m6A-binding protein YTHDF1 [62]. Tumor immune infiltrations in YTHDF1-deficient mice contained higher levels of CD8^+^ cytotoxic T cells and natural killer (NK) cells compared with wild-type mice, indicating that immune surveillance was enhanced in the absence of YTHDF1. So, the absence of YTHDF1 in classical dendritic cells enhanced the cross-presentation of tumor antigens and the cross-primers of CD8^+^ T cells in *vivo* without affecting DCs development or activation [62]. Mechanistically, lysosomal cathepsin, as the main target of YTHDF1 control, affects the cross-priming ability of DCs. Lysosomes contain many types of molecules, such as proteases and antimicrobial peptides, which can be involved in the degradation of permissible pathogens or play a key role in the presentation of MHC class II–restricted antigen presentation [63, 64]. YTHDF1 can identify transcripts that encode lysosomal proteases labeled by m6A, and binds to these transcripts, increasing the translation of lysosomal cathepsin in dendritic cells. Hence, inhibition of cathepsin significantly enhanced the cross-presentation of wild-type dendritic cells. Neoantigen recognition is sufficient to induce a persistent T cell response, which is necessary for complete tumor rejection. Therefore, modulation of YTHDF1 for durable neoantigen-specific immunity may provide new perspectives for enhanced immunotherapeutic response (Fig. 2B) [65].

#### T cells

T cells, as the main group of cellular immunity, play an important role in the killing of tumor cells. In the tumor microenvironment, CD8^+^ T subtypes in T cells can cause killing effect on tumor cells [65], and CD4^+^ T cells can regulate or assist other lymphocytes to function, while Treg cells can inhibit the activation and proliferation of CD4^+^ T cells and CD8^+^ T cells in immune system [66]. Each type of T cell performs its specific function and together build a stable immune system. However, mRNA methylation can control T cell homeostasis and differentiation (Fig. 2C) [67]. After METTL3 was knockout in naive T cells, METTL3-deficient naive T cells showed a decrease ratio in TH1 and TH17 cells, and an increase ratio in TH2 cells compared to wild-type naive T cells, which indicate that m6A modification plays an important role in the differentiation of CD4^+^ T cells. Furthermore, m6A controls the balance of two important signaling pathways that control T cell homeostasis, namely IL-7-mediated JAK-STAT signaling and TCR-mediated ERK/AKT signaling. As the main survival pathway of cells, JAK-STAT signaling can be activated by IL-7 and protect effector / memory T cells from apoptosis [68]. In particular, SOCS protein is a physiological inhibitor of JAK-STAT signaling pathway [69]. SOCS1 negatively regulates IL-7 signaling, while SOCS3 and CISH can inhibit STAT5 phosphorylation and T cell proliferation [69, 70]. SOCS1, SOCS3, and CISH showed higher expression levels in METTL3-deficient primitive T cells. The increased activity of the SOCS family inhibited IL-7-mediated STAT5 activation and T cell homeostasis proliferation and differentiation. Therefore, m6A regulates T cell homeostasis by inducing degradation of transcripts of SOCS family in *vivo* [67]. These findings suggest that m6A modification is critical to the regulation of T cell homeostasis and function.

Similarly, in colorectal cancer and melanoma, METTL3 or METTL14 improved tumor cells to anti-PD-1 therapy by regulating CD8^+^ T cells [71]. CD8^+^ T cells are essential for the control of tumor growth. They produce a variety of cytokines, mainly IFN-γ, TNF-α and other cytokines, which play an important role in anti-tumor immunity [72, 73]. Compared with control tumors, CD8^+^ T cells were significantly increased in METTL3 and METTL14 deficient tumors, and granzyme B expression was significantly enhanced in CD8^+^ T cells. METTL3 and METTL14 deficient cells contain more stable Stat1 and Irf1 mRNAs, and YTHDF2-mediated mRNAs stability also controls the expression of Stat1 and Irf1 genes. Besides, absence of METTL3 or METTL14 makes tumors increase sensitivity to IFN-γ. IFN-γ plays an important role in tumor immunologic surveillance [74]. In the early stage of immune response, IFN-γ mainly comes from NK cells, while in the adaptive stage of immune response, IFN-γ mainly comes from classical CD4^+^ T cells and CD8^+^ T cells [75, 76]. By inducing the production of CXCL9 and CXCL10, these chemokines promote the recruitment of CD8^+^ and CD4^+^ effector T cells and inhibit tumor growth [71]. In addition, CD8^+^ T cells were also regulated by YTHDF in melanoma. Ovalbumin-expressing (OVA) B16 melanoma cells were subcutaneously inoculated into wild-type and YTHDF1-deficient mice. Compared with wild-type mice, YTHDF1-deficient mice contained higher levels of CD8^+^ cytotoxic T cells, and natural killer (NK) cells in tumor immune infiltration. IFN-γ increased significantly in CD8^+^ cytotoxic T cells, and upregulated the expression of PD-L1(ligand of PD1) [62]. Together, these results suggest a mechanism that m6A can modulate the efficacy of immunotherapy not only by regulating the number of CD8^+^ T cells and cytokine production in the tumor microenvironment, but also by achieving high antigen-specific CD8^+^ T cell antitumor response.

### Regulation of m6A on immune evasion

**Fig. 3:**
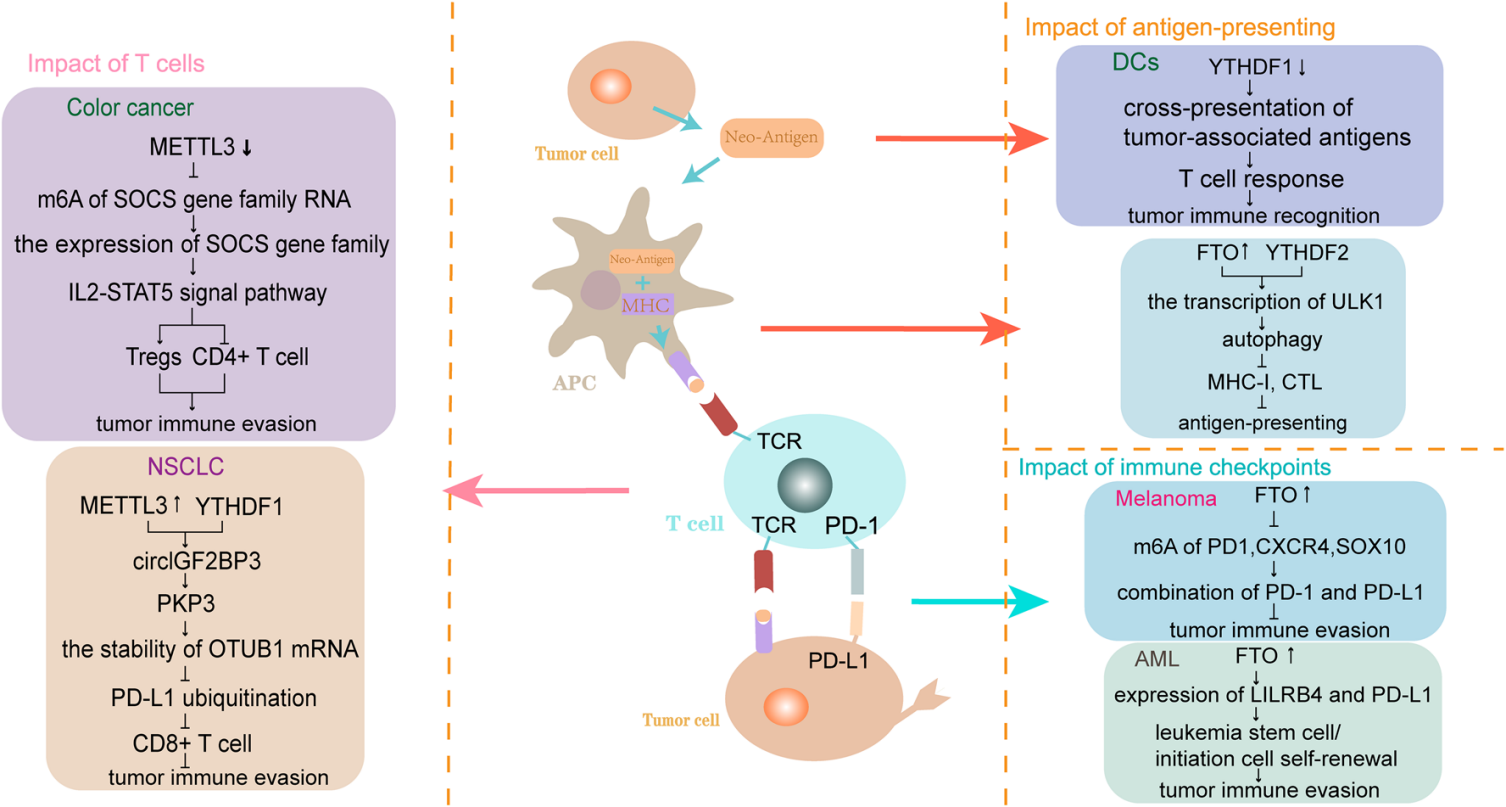
Regulation of m6A on immune evasion. m6A plays an indispensable role in the regulation of immune evasion in various tumors. It functions in several ways: **A** impact of antigen-presenting. **B **impact of T cells. **C **impact of immune checkpoints

Immune evasion of cancer is a major obstacle to the design of effective anticancer treatment strategies. Although researchers have made considerable progress in understanding how cancer evades destructive immunity, it is still a challenge to overcome the immune evasion in tumors [77]. Tumors remain latent in patients for years, when tumor cells can destroy the function of immune system in human body through immunosuppression or loss of target antigen expression, and finally eliminate the immune response [78]. It is at this stage that the tumor escapes from immune surveillance, leading to an apparent clinical cancer. There are many mechanisms that can lead to tumor immune evasion, such as the low immunogenicity of tumor and the decrease of tumor surface antigen expression, the weakening of immune system induced by tumors, the change of TME and so on [79]. More and more studies have shown that m6A plays an indispensable role in the regulation of immune evasion in various tumors and plays an indispensable role in this process (Fig. 3).

#### Impact of antigen-presenting

Defects in major histocompatibility complex(MHC) class I proteins of tumor cells affect antigen presentation and may lead to immune escape [80]. The loss of MHC I antigen presentation not only weakens the ability of natural immune response to control cancer [81], but also weakens the effect of immunotherapy that works by stimulating CD8^+^ T cells [82]. Autophagy can lead to sustained destruction of MHC-I proteins [83]. For example, in pancreatic ductal adenocarcinoma (PDAC), enhanced autophagy flux promotes tumor avoidance by down-regulating MHC-I molecules and by reducing immune recognition induced by CD8^+^ cytotoxic T lymphocytes (CTLs) [84]. Inhibition of autophagy is beneficial to the re-emergence of MHC-I on the surface of malignant cells, while the modification of m6A can regulate autophagy. FTO positively regulates autophagy in an enzyme activity-dependent manner. FTO extended the half-life of ULK1 transcription by reversing m6A mRNA modification of ULK1 transcription, and may affect the stability of ULK1 transcripts in a YTHDF2-dependent manner [85]. While in another study, it was demonstrated that the targets of YTHDF2 are ATG5 and ATG7. When FTO is silenced, YTHDF2 binds more ATG5 and ATG7 transcripts, leading to degradation of mRNA and reduced protein expression, thereby mitigating autophagy [86]. In addition, autophagy regulated by m6A modifications also plays a physiological role in cancer drug resistance. METTL3 can modify FOXO3 mRNA and increase its stability, a process that relies on the assistance of YTHDF1. As a result, deletion of METTL3 in HCC patients destabilizes FOXO3 mRNA and increases drug resistance in HCC by regulating autophagic flux [87]. On the other hand, loss of tumor-associated antigens is another factor that prevents immune recognition. Persistent T cell response is necessary for complete tumor rejection, but loss of tumor-associated antigen cannot induce a persistent T cell response [62]. In classical dendritic cells, YTHDF1 regulates persistent neoantigen-specific immunity. Deletion of YTHDF1 leads to cross presentation of tumor antigens and in *vivo* CD8^+^ T cell cross primers without affecting DCs development or activation [62]. This change undoubtedly enhances the immune system’s surveillance of cancer and it is more conducive to cancer detection and clearance.

#### Impact of T cells

T cells of the body’s immune system can be regulated by m6A and have a direct impact on the immune evasion of tumors. Especially the activity of various types of T cells, like CD8^+^ T cells and Tregs, is a key factor in determining immune evasion [88]. In a recent study, the authors revealed that circIGF2BP3, a novel circRNA, can inhibit the infiltration of CD8^+^ tumor infiltrating lymphocyte(TIL) and mediate immune evasion in non-small cell lung cancer(NSCLC) [89], which process is correlated with m6A modification. In NSCLC cells, METTL3-mediated m6A modification of circIGF2BP3 promotes circIGF2BP3 upregulation by promoting its back-splicing and circularization in a manner dependent on YTHDC1. CircIGF2BP3 stabilizes OTUB1 mRNA by competitively upregulating PKP3 expression, and then reduced PD-L1 ubiquitination and subsequent proteasome degradation, leading to CD8^+^ T cell-mediated immune escape [89]. The eventual formation of immune evasion is closely related to the changes of T cells mediated by m6A in the tumor microenvironment. And this process was also involved and modulated by immune checkpoints, suggesting that immune checkpoints play a role in immune evasion at the same time. Similarly, in colon cancer, regulatory T cells (Tregs), which inhibit immunity and promote tumorigenesis, were up-regulated and the infiltration of activated memory T cells CD4 (CD4^+^ T cells) was down-regulated in the group with high expression of the immune gene CD34/CD276. Regulatory T cell is a key factor in tumor immune escape. The hallmark of Treg cells is the transcription factor Foxp3, while Treg cells also express higher IL-2 receptors [90, 91]. IL-2 receptors can be regulated by the SOCS family and activate STAT5 [92]. When METTL3 in Treg cells is depleted, it will cause the increase of SOCS gene family mRNA, thus inhibiting the IL2-STAT5 signaling pathway, and finally maintain the Treg inhibitory function [93]. Exploring the mechanism of T cell-mediated immune evasion and discovering more about its relationship with m6A modifications will facilitate immunotherapy of tumors.

#### Impact of immune checkpoints

Immune checkpoints are a kind of immunosuppressive molecules, which are expressed on immune cells and can regulate the degree of immune activation [94]. They play an important role in preventing the occurrence of autoimmunity [95]. Immune checkpoint molecules keep the immune system within a normal range when it is activated, so that the immune system is not overactivated. Their abnormal expression and function are one of the important reasons for tumor immune escape. Programmed Cell Death Protein 1(PD1) is a typical representative of immune checkpoint inhibitors. PD1 inhibits the function of T lymphocytes by binding to the ligand PDL1/PDL2, thereby inhibiting the autoimmune response [96, 97]. In melanoma, the modification of m6A in RNA can reduce the proliferation and viability of tumor cells, suggesting that m6A has anti-tumor effect in melanoma. FTO can reduce the enrichment of m6A in key melanoma promoting genes, including PD-1 (PDCD1), CXCR4 and SOX10, and maintain their mRNA stability. By enhancing the binding of PD1 to the ligand PDL1/PDL2, it reduced the immune regulatory response of melanoma, and then promoted the immune escape of tumor [98]. Furthermore, down-regulation of ALKBH5 was associated with positive Pembrolizumab or Nivolumab response to PD-1 blockade in melanoma patients, further demonstrating the role of m6A-modified and immune checkpoints in tumor immune evasion [99]. FTO was found to regulate the expression of PD-L1 in leukemia. Gene deletion of FTO significantly attenuates leukemia stem cell/initiation cell self-renewal and reprogramming immune response by inhibiting the expression of immune checkpoint genes (especially LILRB4) [100]. FTO suppression makes leukemia cells sensitive to T cell toxicity and overcomes hypomethylation induced immune evasion. This provides help for the treatment of leukemia. Similarly, osteopontin (OPN), expressed by tumor cells in colon cancer, is an immune checkpoint that is a ligand for CD44 on the surface of T cells, and by binding to CD44 can effectively suppress T cell activity, resulting in enhanced cancer immune tolerance [101]. Thus, when the suppressor of OPN, interferon regulatory factor 8 (IRF8) is reduced, OPN expression is significantly increased, leading to immune evasion of cancer.

### Immunotherapy

In the previous part of the article, we have discussed various mechanisms of immune evasion and immune regulation in cancer. Currently, common cancer treatment methods in clinical practice mainly include chemotherapy and radiotherapy, immunotherapy, targeted therapy and surgical treatment [102]. Each of these methods has its own advantages and limitations, but one of the most promising treatments is immunotherapy. Cancer immunotherapy is a therapeutic method to control and remove tumors by restarting and maintaining the tumor-immune cycle and restoring the normal anti-tumor immune response of the body, including immune checkpoint blockade [103], cell therapy [104], therapeutic antibodies, cancer vaccine [105] and so on.

#### Immune checkpoint blockade and m6A inhibitors

Among many immunotherapy strategies, immune checkpoint blockade shows significant benefits. As a molecular target, immune checkpoint blockade enhances anti-tumor immunity by blocking inherent immune down-regulating factors, such as cytotoxic t-lymphocyte antigen 4 (CTLA-4) [106]and programmed cell death 1 (PD-1) or their ligands, programmed cell death ligand 1 (PD-L1) (Table 1) [107]. Programmed Cell Death Protein 1(PD1) is a typical representative of immune checkpoint [108]. PD1 inhibits the function of T lymphocytes by binding to the ligand PD-L1/PD-L2, thereby inhibiting the autoimmune response [109]. A variety of tumors can be improved by inhibiting PD1/PD-L1, and PD-1 inhibitors Pembrolizumab and Nivolumab have been approved by the FDA for advanced melanoma and non-small cell lung cancer [110].

**Table 1:** Immune checkpoints, their ligands distribution and antibody

Immune checkpoint	Ligand	Distribution of ligand	Antibody	Antibody type	Cancer
CTLA-4	CD80(B7-1) CD86(B7-2)	APCs	Ipilimumab	Human IgG1	Melanoma
PD-1	PD-L1(B7-H1) PD-L2(B7-DC)	Hematopoietic cells Non-hematopoietic cells Tumor cells	Pembrolizumab Nivolumab Atezolizumab	Human IgG4 Human IgG4 Human IgG1	Melanoma NSCLC
LAG-3	MHC-II Galectin-3 LSECtin FGL-1	APCs Tumor cells	GSK2831781	Human IgG	Melanoma Colon adenocarcinoma Ovarian cancer
Tim3	Galectin-9 Phosphatidylserine CEACAM1 HMGB1	Hematopoietic cells Apoptotic cells Tumor cells	TSR-022 LY3321367 MBG453	Human IgG4 Human IgG1 Human IgG4	Liver cancer Solid tumor AML
TIGIT	CD155(PVR) CD112(PVRL2)	APCs, T cells Non-hematopoietic cells, Tumor cells	Tiragolumab AB-154 BMS-986,207	Human IgG1 Human IgG1 Human IgG1	NSCLC Solid tumor

Interestingly, m6A modification is involved in the regulation of PD1, PD-L1 and affects the tumor response to anti-PD-1 therapy. For example, in melanoma, all of METTL3/14, ALKBH5 and FTO can modulate tumor response to anti-PD-1 therapy in mice. When Mettl3/14 was inhibited, the modification of mRNA in the tumor was reduced, leading to an increase in CD8^+^ T cells, and enhanced the response to anti-PD-1therapy by a series of signaling pathways [71]. The low expression of demethylase ALKBH5 was associated with better survival in patients with melanoma. ALKBH5 regulates the expression of Mct4/Slc16a3 and the content of lactic acid in tumor microenvironment, as well as the composition of Tregs and bone marrow-derived suppressor cells. When a specific ALKBH5 inhibitor ALK-04 was used, the expression of ALKBH5 was down-regulated and the effect of anti-PD-1 therapy was enhanced, indicating that ALKBH5 can be used as a potential therapeutic target for melanoma [99]. Similarly, inhibition of FTO can reduce tumor resistance to PD-1 therapy [98]. If inhibition of FTO is combined with anti-PD-1 blocking immunotherapy, the therapeutic effect is more pronounced. In addition, OVA-expressing B16 melanoma cell skins were inoculated into wild-type and YTHDF1^−/−^ mice to construct models. PD-L1 checkpoint blockade was more effective in YTHDF1^−/−^ mice than in wild-type mice. This was due to increased IFN-γ expression in CD8^+^ T cells of YTHDF1^−/−^ mice, and IFN-γ signaling up-regulated PD-L1 expression [62]. This suggested that the combination of YTHDF1 inhibitor and checkpoint blockade may be a potential novel therapeutic strategy to improve the outcome of patients with low response to checkpoint blockade. Therefore, the rational use of m6A inhibitors has great potential and prospect for tumor regulation and treatment.

In fact, m6A inhibitors play a significant role in cancer treatment not only because of its anti-tumor immunity, but also because of its anti-tumor activity (Table 2). In leukemia, the use of CS1 and CS2 targeting FTO increases the sensitivity of AML cells to T cell toxicity and overcomes HMA-induced immune escape, thereby achieving therapeutic effects [100]. FB23-2 can also inhibit FTO to suppress proliferation and promote the differentiation of AML [111], and Rhein can inhibit FTO to overcome tyrosine kinase inhibitor [112]. Besides, FTO inhibitor MA/MA2 can suppress the growth and self-renewal of GSCs in GBM [113]. It can be seen that there are many kinds of FTO-targeting inhibitors and their application range is also very wide. In addition to FTO-targeting inhibitors, the latest research shows that a new small-molecule inhibitior of METTL3 against AML. STM2457 is a highly specific inhibitor of catalytic activity for METTL3, but has no significant inhibitory effect on other RNA methyltransferases. Pharmacological inhibition of METTL3 in *vivo* can weaken the proliferation of AML stem cells or leukemia, inhibit the expansion of AML, and lead to transplantation damage and prolongation of survival time of AML mice, but there was no such expression in normal non-leukemic hematopoietic cells [114]. While SPI1 can also inhibit AML cell survival by regulating METTL14 [115]. In addition to m6A inhibitors, some regulators targeting m6A can also exert anti-tumor effects. In colon cancer, the presence of LINRIS (Long Intergenic Noncoding RNA for IGF2BP2 Stability) in large amounts maintains the stability of “reader” IGF2BP2, because the autophagy-lysosome pathway is blocked [116]. When LINRIS is knocked down, the amount of IGF2BP2 is reduced and downstream effect—the MYC-mediated glycolysis in CRC cancer cells decreases, leading to slower tumor growth. R-2HG, as a metabolite produced by mutant isocitrate dehydrogenases (IDHs), can inhibit FTO activity to increase the accumulation of m6A modified m6A in MYC transcripts of sensitive leukemia cells, resulting in decreased stability of MYC mRNA and downregulation of MYC signaling pathway [117]. This regulation helps to inhibit the proliferation of cancer. Interestingly, the latest research result shows that R-2HG also promotes its anti-tumor activity by participating in the glycolysis pathway. PFKP and LDHB play an important role in glycolysis and tumorigenesis of leukemia. They are regulated by the R-2HG/FTO axis in an m6A-dependent manner. R-2HG can block the post-transcriptional upregulation of PFKP and LDHB mediated by FTO/m6A, thus attenuating aerobic glycolysis in sensitive(IDH-wildtype) leukemic cells and inhibiting the occurrence of leukemia [118]. This conclusion also indicates the therapeutic potential of m6A targeting tumor transcriptome and metabolism.

**Table 2:** m6A inhibitors have different targets and mechanisms

Cancer/Disease	Drug	Target	Role	Mechanism
AML	STM2457	METTL3	Inhibitor	Weaken the proliferation of AML stem cells or leukemia
	SPI1	METTL14	Regulator	Promote terminal myeloid differentiation of normal HSPCs/AML Inhibit AML cell survival
	CS1/CS2	FTO	Inhibitor	Increase the sensitivity of AML cells to T cell toxicity
	R-2HG	FTO	Inhibitor	Inhibit the proliferation of AML and weaken aerobic glycolysis in sensitive leukemic cells
	FB23-2	FTO	Inhibitor	Suppress proliferation and promote the differentiation of AML
	Rhein	FTO	Inhibitor	Overcome tyrosine kinase inhibitor resistance
GBM	MA/MA2	FTO	Inhibitor	Suppress the growth and self-renewal of GSCs
Melanoma	ALK-04	ALKBH5	Inhibitor	Regulate the express of Mct4/Scl16a3 and lactic acid
BC	MO-I-500	FTO	Inhibitor	Suppress survival of BC cells by decreasing IRX3 proteins
LUAD	PKE Sorafenib	YTHDC2	Regulator	Suppress SLC7A11-dependent antioxidant function
Testicular damage	MEHP	FTO	Inhibitor	Regulate reduction of testosterone and increase of apoptosis

In summary, the above findings provide us with a new idea that the development of specific inhibitors targeting m6A regulators contributes to anti-tumor therapy. On the one hand, studies have shown that most m6A regulators are aberrantly highly expressed in tumor cells, so targeted inhibition of these regulators can directly inhibit tumor cell activity and further improve treatment outcomes and patient prognosis; on the other hand, the regulation of m6A is closely related to the development and function of immune cells. Targeted modulation of m6A can enhance the function and infiltration of immune cells in the tumor microenvironment and enhance the tumor response to anti-PD-1 therapy through a number of signaling pathways. It can be seen that the use of specific inhibitors targeting m6A regulatory factors can combine anti-tumor activity and anti-tumor immunity to play a comprehensive therapeutic effect. Attempts to combine m6A inhibitors and immune checkpoint blockade may be a new breakthrough in tumor immunotherapy.

In addition to immunotherapy targeting PD-1, Cytotoxic T-lymphocyte antigen 4(CTLA-4) is also a common target for tumor immunotherapy. CTLA-4 is a transmembrane protein expressed on the surface of activated T cells. CTLA-4 has two ligands: B7-1(CD80) and B7-2(CD86). When it binds to ligands, it produces inhibitory signals of T cells and inhibits the initiation of T cell immune responses, resulting in a decrease in activated T cells and preventing the formation of memory T cells [119]. Blocking CTLA-4 can restore the activity of T cells and prolong the survival time of memory T cells. This allows the body to further strengthen the immune function of tumor cells, resulting in increased tumor control rate [120]. Therefore, CTLA-4 inhibitors also play an important role in tumor immunity, especially in melanoma. Currently, CTLA-4 inhibitor Ipilimumab has been approved by FDA for the adjuvant treatment of stage III melanoma and the treatment of advanced melanoma [121], and clinical studies of Ipilimumab and Telimomab in kidney cancer, prostate cancer and lung cancer have been widely carried out. As for other immune checkpoints, such as LMTK3, LAG-3/CD223, TIM3, are also related to the activity and function of T lymphocytes and can be used as targets for immunotherapy (Table 1). For example, LMTK3 is a client protein of heat shock protein 90 (HSP90). It plays a cancer-promoting role in breast cancer, and C28, a LMTK3 small-molecule inhibitor, can promote proteasome-mediated LMTK3 degradation. The inhibition of C28 reduced the proliferation of NCI-60 tumor cell line and increased the apoptosis of breast cancer cells [122]. LAG3(Lymphocyte Activation Gene3) is mainly expressed in activated T lymphocytes, B lymphocytes, NK cells and plasma cell-like dendritic cells, and negatively regulates T cell function. LAG-3 selectively upregulates the expression of CD4 on the surface of Treg, and inhibition or knockout of LAG-3 will relieve the inhibitory function of Treg on T cells [123]. It is worth noting that when the interaction between LAG3 and ligand FGL1 is blocked by monoclonal antibodies, the T cell response in the tumor is enhanced and the tumor volume decreases, which provides a new idea for targeted immunotherapy [124]. TIM3 (T cell immunoglobulin-3), a receptor protein of the TIM family, is expressed on T cells, Treg cells, dendritic cells, NK cells and monocytes. TIM3 has a variety of ligands, which can inhibit the activity of effector T cells and induce peripheral tolerance [125]. TIGIT (T Cell Immunoreceptor With Ig And ITIM Domains) is a receptor of immunoglobulin superfamily, which can limit acquired immunity and innate immunity. Other ICBs combined with TIGIT blocking can enhance the anti-tumor immune response, such that blocking TIGIT and PD-1 together can enhance the proliferation of CD8^+^ T cells and protect memory T cells [126]. Although the relationship between these immune checkpoints and m6A has not been deeply studied, their potential impact on tumor immunotherapy cannot be ignored.

#### Cell therapy and m6A

American biologist George Daley once said: “If the 20th century is the era of drug therapy, then the 21st century will be the era of cell therapy.“ Cell therapy can separate immunoreactive cells from tumor patients, amplify and identify their functions in *vitro*, and then infuse them back to patients, so as to kill tumor cells directly or stimulate human immune response to kill tumor cells. When the immunogenicity of cancer is poor, the use of immune checkpoint therapy alone may not achieve the desired effect [127]. If properly combined with cell therapy, immunotherapy for these poorly immunogenic types of cancer will be possible and may enhance the response of tumors that are already responsive to immune checkpoint therapy [104]. ACT mainly includes TIL, LAK, CIK, DC, NK, TCR-T, CAR-T and so on. As the “star” of tumor immune cell therapy, CAR-T cell therapy has shown satisfying targeting, lethality and persistence in clinical trials [128], which provides a new solution for immune cell therapy and shows great development potential and application prospects. CAR-T is a specific cellular immunotherapy [129], which is different from non-specific cellular immunotherapy such as CIK, DC-CIK, NK and so on. CAR combines the antigen binding domain with the additional costimulatory domain from CD28, OX40 and CD137 receptors and the signal domain of the TCR chain, which makes T cells have the specificity of cytotoxicity and antibody recognition at the same time [130]. In the second part of the article, we introduced that MHC I protein deficiency in tumor cells can affect antigen presentation and may lead to immune escape. CAR recognition overcomes the limitation that TCR needs MHC expression and recognition, and shows the anti-tumor advantage of CAR T cells. CD19 is a kind of B cell antigen expressed on the surface of normal and malignant B cells. The second-generation CAR T cells target CD19 and encode a costimulatory domain, showing a lasting response and strong curative effect on B-cell malignant tumors [131, 132]. However, although CAR T cells provide new ideas for the treatment of hematological malignant tumors, there are still many difficulties in successfully applying these treatments to solid tumors. But as discussed above, m6A modification is closely related to the function of T cells. For example, METTL3 can regulate the dynamic balance and differentiation of T cells, and YTHDF1 can regulate the number of CD8^+^ T cells infiltrated in tumor microenvironment in mice. Therefore, we make a conjecture that attempting to regulate m6A modification in CAR T cells may be a satisfying strategy to enhance tumor immunotherapy. Maybe with the continuous development of cell engineering and gene editing, this idea is expected to be gradually improved and applied to many types of tumor therapy.

In addition to CAR-T cell therapy, adoptive immunotherapy based on NK cells is also an important part of cell therapy [133]. Song et al. found that in mice, down-regulation of METTL3 expression in NK cells leads to a decrease in SHP-2 activity, which contributes to a decrease in NK cell response to IL-15, thereby affecting the proliferation and differentiation of NK cells [134]. This finding reveals the regulation of m6A modification on NK cells and provides a potential approach to address the problem of reduced proliferation efficiency of NK cells in *vitro*, which slows down the progress of cell therapy.

#### m6A and other targets

In addition to immune checkpoints and cell therapy, some regulatory cells related to tumor immunity, or related products involved in tumor immune escape, can be used as targets to enhance the effect of tumor immunotherapy [77]. For example, Tregs in tumor microenvironment inhibits the immune response of the body to tumor, which is a factor hindering immunotherapy [66]. It can be regulated by m6A to change the response of tumor to immunotherapy. The knockout of ALKBH5 can reduce the infiltration of Tregs in melanoma and enhance the response to anti-PD-1 therapy [99]; MDSCs can induce Tregs production, thus promote tumor angiogenesis and metastasis, so it can also be a target for improving treatment [135]; the polarization of macrophages can affect tumor formation, so altering macrophage polarization in the tumor microenvironment through m6A modification can also provide help in treating cancer [51, 53]; Tumor autophagy leads to tumor immune escape. m6A can control autophagy to improve the continuous destruction of MHC-I protein and the occurrence of tumor immune escape, and then enhance the immune response of tumor to the body. To sum up, m6A is widely involved in all kinds of tumor immune regulation, and has great work and potential in tumor immunotherapy.

## Discussion and perspectives

In recent years, the roles of m6A modification in different diseases and its molecular mechanism has become a research hotspot. m6A plays a dual role in cancer, like a double-edged sword. On the one hand, m6A can regulate the expression of oncogenes or tumor suppressor genes, thus affecting the progression of cancer; on the other hand, regulating the level of m6A and the expression and activity of m6A enzyme can affect the roles of m6A in cancer. In this review, we discuss the immunomodulatory effect of m6A on tumor from three stages: “tumor growth”-immune escape, “tumor control”- immune surveillance and “tumor elimination”-immunotherapy. In terms of immune evasion, we summarized that m6A can enhance the effect of antigen presentation, and can also modulate the immune activity of effector T cells after antigen presentation, thereby managing cancer evasion. When cancer destabilizes the body’s immune system, m6A can modulate various immune cells in the tumor microenvironment to kill the cancer. By mediating macrophage polarization, DC maturation and T cell homeostasis and differentiation, m6A can exert effective control over tumor growth. Most importantly, m6A provides a new direction for the treatment of tumors. The levels of m6A affect the tumor response to immune checkpoint blockade therapy. Therefore, the combined use of m6A inhibitors and immune checkpoint blockade can be more favorable to patient treatment and prognosis. In fact, m6A modification can also improve the drug resistance of cancer cells and make other anti-cancer drugs work better, but this mechanism we do not detail here [136]. In conclusion, an in-depth understanding of the mechanism of m6A in immune regulation will help to develop new and effective treatment strategies, which can be used in the clinical treatment and prognosis of cancer patients.

## Data Availability

Not applicable.

## Abbreviations

m6A: N6-methyladenosine
UTR: Untranslated terminal region
NcRNA: Noncoding RNA
METTL: Methyltransferase-like
WTAP: Wilms tumor-associated protein
ALKBH: ALKB Homolog
LncRNA: Long noncoding RNA
RBM15: RNA binding motif protein 15
FTO: Fat mass and obesity-associated protein
STAT1: Signal transducer and activator of transcription 1
PPAR-γ: Peroxisome proliferator-activated receptorγ
SOCS1: Suppressor of cytokine signaling 1
DCs: Dendritic cells
TIRAP: Toll-interleukin 1 receptor (TIR) domain containing adaptor protein
Stat1: Signal transducer and activator of transcription 1
PDAC: Pancreatic ductal adenocarcinoma
ULK1: Unc-51 like autophagy activating kinase 1
FOXO3: Forkhead box O3
CircRNA: Circular RNA
NSCLC: Non-small cell lung carcinoma
OTUB1: OTU deubiquitinase, ubiquitin aldehyde binding 1
PD-1: Programmed cell death protein 1
PD-L1: Programmed cell death ligand 1
CTLA-4: Cytotoxic t-lymphocyte antigen 4
AML: Acute myelocytic leukemia
GBM: Glioblastoma multiforme

## References

[1] Boccaletto P, Machnicka MA, Purta E, Piatkowski P, Baginski B, Wirecki TK, de Crecy-Lagard V, Ross R, Limbach PA, Kotter A (2018). MODOMICS: a database of RNA modification pathways. 2017 update. Nucleic Acids Res.

[2] Yue Y, Liu J, He C (2015). RNA N6-methyladenosine methylation in post-transcriptional gene expression regulation. Genes Dev.

[3] Jia G, Fu Y, He C (2013). Reversible RNA adenosine methylation in biological regulation. Trends Genet..

[4] Sun T, Wu R, Ming L (2019). The role of m6A RNA methylation in cancer. Biomed Pharmacother.

[5] Dominissini D, Moshitch-Moshkovitz S, Schwartz S, Salmon-Divon M, Ungar L, Osenberg S, Cesarkas K, Jacob-Hirsch J, Amariglio N, Kupiec M (2012). Topology of the human and mouse m6A RNA methylomes revealed by m6A-seq. Nature.

[6] Meyer KD, Saletore Y, Zumbo P, Elemento O, Mason CE, Jaffrey SR (2012). Comprehensive analysis of mRNA methylation reveals enrichment in 3’ UTRs and near stop codons. Cell..

[7] Ke S, Alemu EA, Mertens C, Gantman EC, Fak JJ, Mele A, Haripal B, Zucker-Scharff I, Moore MJ, Park CY (2015). A majority of m6A residues are in the last exons, allowing the potential for 3’ UTR regulation. Genes Dev.

[8] Gilbert WV, Bell TA, Schaening C, Messenger (2016). RNA modifications: Form, distribution, and function. Science..

[9] Lipshitz HD, Claycomb JM, Smibert CA (2017). Post-transcriptional regulation of gene expression. Methods.

[10] Roignant JY, Soller M (2017). m(6)A in mRNA: An Ancient Mechanism for Fine-Tuning Gene Expression. Trends Genet..

[11] Pan Y, Ma P, Liu Y, Li W, Shu Y (2018). Multiple functions of m(6)A RNA methylation in cancer. J Hematol Oncol.

[12] Bokar JA, SO MER-S, PolLudwiczakayes R, Narayann P, Rottman F (1994). Characterization and partial purification of mRNA NG-Adenosine methyltransferase from HeLa Cell Nucle. J Biol Chem.

[13] Bokar JA, Shambaugh ME, Polayes D, Matera AG, Rottman FM (1997). Purification and cDNA cloning of the AdoMet-binding subunit of the human mRNA (N6-adenosine)-methyltransferase. RNA.

[14] Wang X, Feng J, Xue Y, Guan Z, Zhang D, Liu Z, Gong Z, Wang Q, Huang J, Tang C (2016). Structural basis of N(6)-adenosine methylation by the METTL3-METTL14 complex. Nature..

[15] Wang P, Doxtader KA, Nam Y (2016). Structural Basis for Cooperative Function of Mettl3 and Mettl14 Methyltransferases. Mol Cell..

[16] Liu J, Yue Y, Han D, Wang X, Fu Y, Zhang L, Jia G, Yu M, Lu Z, Deng X (2014). A METTL3-METTL14 complex mediates mammalian nuclear RNA N6-adenosine methylation. Nat Chem Biol..

[17] Pendleton KE, Chen B, Liu K, Hunter OV, Xie Y, Tu BP, Conrad NK (2017). The U6 snRNA m(6)A Methyltransferase METTL16 Regulates SAM Synthetase Intron Retention. Cell..

[18] Schwartz S, Mumbach MR, Jovanovic M, Wang T, Maciag K, Bushkin GG, Mertins P, Ter-Ovanesyan D, Habib N, Cacchiarelli D (2014). Perturbation of m6A writers reveals two distinct classes of mRNA methylation at internal and 5’ sites. Cell Rep..

[19] Patil DP, Chen CK, Pickering BF, Chow A, Jackson C, Guttman M, Jaffrey SR (2016). m(6)A RNA methylation promotes XIST-mediated transcriptional repression. Nature..

[20] Jia GF, Fu Y, Zhao X, Dai Q, Zheng GQ, Yang Y, Yi CQ, Lindahl T, Pan T, Yang YG (2011). N6-Methyladenosine in nuclear RNA is a major substrate of the obesity-associated FTO. Nat Chem Biol.

[21] Zheng G, Dahl JA, Niu Y, Fedorcsak P, Huang CM, Li CJ, Vagbo CB, Shi Y, Wang WL, Song SH (2013). ALKBH5 is a mammalian RNA demethylase that impacts RNA metabolism and mouse fertility. Mol Cell..

[22] Lee M, Kim B, Kim VN (2014). Emerging roles of RNA modification: m(6)A and U-tail. Cell..

[23] Ueda Y, Ooshio I, Fusamae Y, Kitae K, Kawaguchi M, Jingushi K, Hase H, Harada K, Hirata K, Tsujikawa K (2017). AlkB homolog 3-mediated tRNA demethylation promotes protein synthesis in cancer cells. Sci Rep.

[24] Liu C, Yang Z, Li R, Wu Y, Chi M, Gao S, Sun X, Meng X, Wang B (2021). Potential roles of N6-methyladenosine (m6A) in immune cells. J Transl Med..

[25] Dai D, Wang H, Zhu L, Jin H, Wang X (2018). N6-methyladenosine links RNA metabolism to cancer progression. Cell Death Dis..

[26] Zhou Z, Lv J, Yu H, Han J, Yang X, Feng D, Wu Q, Yuan B, Lu Q, Yang H (2020). Mechanism of RNA modification N6-methyladenosine in human cancer. Mol Cancer..

[27] Yi YC, Chen XY, Zhang J, Zhu JS (2020). Novel insights into the interplay between m(6)A modification and noncoding RNAs in cancer. Mol Cancer.

[28] Wang X, Zhao BS, Roundtree IA, Lu Z, Han D, Ma H, Weng X, Chen K, Shi H, He C (2015). N(6)-methyladenosine Modulates Messenger RNA Translation Efficiency. Cell..

[29] Huang T, Liu Z, Zheng Y, Feng T, Gao Q, Zeng W (2020). YTHDF2 promotes spermagonial adhesion through modulating MMPs decay via m(6)A/mRNA pathway. Cell Death Dis..

[30] Du H, Zhao Y, He J, Zhang Y, Xi H, Liu M, Ma J, Wu L (2016). YTHDF2 destabilizes m(6)A-containing RNA through direct recruitment of the CCR4-NOT deadenylase complex. Nat Commun..

[31] Wang X, Lu Z, Gomez A, Hon GC, Yue Y, Han D, Fu Y, Parisien M, Dai Q, Jia G (2014). N6-methyladenosine-dependent regulation of messenger RNA stability. Nature..

[32] Li A, Chen YS, Ping XL, Yang X, Xiao W, Yang Y, Sun HY, Zhu Q, Baidya P, Wang X (2017). Cytoplasmic m(6)A reader YTHDF3 promotes mRNA translation. Cell Res..

[33] Shi H, Wang X, Lu Z, Zhao BS, Ma H, Hsu PJ, Liu C, He C (2017). YTHDF3 facilitates translation and decay of N(6)-methyladenosine-modified RNA. Cell Res..

[34] Xiao W, Adhikari S, Dahal U, Chen YS, Hao YJ, Sun BF, Sun HY, Li A, Ping XL, Lai WY (2016). Nuclear m(6)A Reader YTHDC1 Regulates mRNA Splicing. Mol Cell..

[35] Roundtree IA, Luo GZ, Zhang Z, Wang X, Zhou T, Cui Y (2017). YTHDC1 mediates nuclear export of N(6)-methyladenosine methylated mRNAs. Elife.

[36] Shima H, Matsumoto M, Ishigami Y, Ebina M, Muto A, Sato Y, Kumagai S, Ochiai K, Suzuki T, Igarashi K (2017). S-Adenosylmethionine Synthesis Is Regulated by Selective N(6)-Adenosine Methylation and mRNA Degradation Involving METTL16 and YTHDC1. Cell Rep.

[37] Hsu PJ, Zhu Y, Ma H, Guo Y, Shi X, Liu Y, Qi M, Lu Z, Shi H, Wang J (2017). Ythdc2 is an N(6)-methyladenosine binding protein that regulates mammalian spermatogenesis. Cell Res..

[38] Wojtas MN, Pandey RR, Mendel M, Homolka D, Sachidanandam R, Pillai RS (2017). Regulation of m(6)A Transcripts by the 3’-->5’ RNA Helicase YTHDC2 Is Essential for a Successful Meiotic Program in the Mammalian Germline. Mol Cell..

[39] Wang S, Lv W, Li T, Zhang S, Wang H, Li X, Wang L, Ma D, Zang Y, Shen J (2022). Dynamic regulation and functions of mRNA m6A modification. Cancer Cell Int..

[40] Huang H, Weng H, Sun W, Qin X, Shi H, Wu H, Zhao BS, Mesquita A, Liu C, Yuan CL (2018). Recognition of RNA N(6)-methyladenosine by IGF2BP proteins enhances mRNA stability and translation. Nat Cell Biol..

[41] Zhao BS, Roundtree IA, He C (2017). Post-transcriptional gene regulation by mRNA modifications. Nat Rev Mol Cell Biol..

[42] Epelman S, Lavine KJ, Randolph GJ (2014). Origin and functions of tissue macrophages. Immunity..

[43] Hirayama D, Iida T, Nakase H (2017). The phagocytic function of macrophage-enforcing innate immunity and tissue homeostasis. Int J Mol Sci.

[44] Murray PJ, Allen JE, Biswas SK, Fisher EA, Gilroy DW, Goerdt S, Gordon S, Hamilton JA, Ivashkiv LB, Lawrence T (2014). Macrophage activation and polarization: nomenclature and experimental guidelines. Immunity..

[45] Shapouri-Moghaddam A, Mohammadian S, Vazini H, Taghadosi M, Esmaeili SA, Mardani F, Seifi B, Mohammadi A, Afshari JT, Sahebkar A (2018). Macrophage plasticity, polarization, and function in health and disease. J Cell Physiol..

[46] Koo SJ, Garg NJ (2019). Metabolic programming of macrophage functions and pathogens control. Redox Biol.

[47] Canton M, Sanchez-Rodriguez R, Spera I, Venegas FC, Favia M, Viola A, Castegna A (2021). Reactive Oxygen Species in Macrophages: Sources and Targets. Front Immunol.

[48] Liu P, Peng J, Han GH, Ding X, Wei S, Gao G, Huang K, Chang F, Wang Y (2019). Role of macrophages in peripheral nerve injury and repair. Neural Regen Res.

[49] Aminin D, Wang YM (2021). Macrophages as a “weapon” in anticancer cellular immunotherapy. Kaohsiung J Med Sci..

[50] Boutilier AJ, Elsawa SF. Macrophage polarization states in the tumor microenvironment. Int J Mol Sci. 2021; 22. 10.3390/ijms22136995 PMC8268869 34209703

[51] Liu Y, Liu Z, Tang H, Shen Y, Gong Z, Xie N, Zhang X, Wang W, Kong W, Zhou Y (2019). The N(6)-methyladenosine (m(6)A)-forming enzyme METTL3 facilitates M1 macrophage polarization through the methylation of STAT1 mRNA. Am J Physiol Cell Physiol.

[52] Lim WS, Timmins JM, Seimon TA, Sadler A, Kolodgie FD, Virmani R, Tabas I (2008). Signal transducer and activator of transcription-1 is critical for apoptosis in macrophages subjected to endoplasmic reticulum stress in vitro and in advanced atherosclerotic lesions in vivo. Circulation.

[53] Gu X, Zhang Y, Li D, Cai H, Cai L, Xu Q (2020). N6-methyladenosine demethylase FTO promotes M1 and M2 macrophage activation. Cell Signal.

[54] Schultze JL, Schmidt SV (2015). Molecular features of macrophage activation. Semin Immunol..

[55] Lawrence T, Natoli G (2011). Transcriptional regulation of macrophage polarization: enabling diversity with identity. Nat Rev Immunol..

[56] Huangfu N, Zheng W, Xu Z, Wang S, Wang Y, Cheng J, Li Z, Cheng K, Zhang S, Chen X (2020). RBM4 regulates M1 macrophages polarization through targeting STAT1-mediated glycolysis. Int Immunopharmacol.

[57] Du J, Liao W, Liu W, Deb DK, He L, Hsu PJ, Nguyen T, Zhang L, Bissonnette M, He C (2020). N(6)-Adenosine Methylation of Socs1 mRNA Is Required to Sustain the Negative Feedback Control of Macrophage Activation. Dev Cell..

[58] Qian C, Cao X (2018). Dendritic cells in the regulation of immunity and inflammation. Semin Immunol..

[59] Chen S, Fang L, Guo W, Zhou Y, Yu G, Li W, Dong K, Liu J, Luo Y, Wang B (2018). Control of Treg cell homeostasis and immune equilibrium by Lkb1 in dendritic cells. Nat Commun..

[60] Wang H, Hu X, Huang M, Liu J, Gu Y, Ma L, Zhou Q, Cao X (2019). Mettl3-mediated mRNA m(6)A methylation promotes dendritic cell activation. Nat Commun..

[61] Horng T, Barton GM, Medzhitov R (2001). TIRAP: an adapter molecule in the Toll signaling pathway. Nat Immunol.

[62] Han D, Liu J, Chen C, Dong L, Liu Y, Chang R, Huang X, Liu Y, Wang J, Dougherty U (2019). Anti-tumour immunity controlled through mRNA m(6)A methylation and YTHDF1 in dendritic cells. Nature..

[63] Lennon-Dumenil AM, Bakker AH, Maehr R, Fiebiger E, Overkleeft HS, Rosemblatt M, Ploegh HL, Lagaudriere-Gesbert C (2002). Analysis of protease activity in live antigen-presenting cells shows regulation of the phagosomal proteolytic contents during dendritic cell activation. J Exp Med..

[64] Guermonprez P, Valladeau J, Zitvogel L, Thery C, Amigorena S (2002). Antigen presentation and T cell stimulation by dendritic cells. Annu Rev Immunol..

[65] Fu C, Jiang A (2018). Dendritic Cells and CD8 T Cell Immunity in Tumor Microenvironment. Front Immunol.

[66] Sakaguchi S, Mikami N, Wing JB, Tanaka A, Ichiyama K, Ohkura N (2020). Regulatory T Cells and Human Disease. Annu Rev Immunol.

[67] Li HB, Tong J, Zhu S, Batista PJ, Duffy EE, Zhao J, Bailis W, Cao G, Kroehling L, Chen Y (2017). m(6)A mRNA methylation controls T cell homeostasis by targeting the IL-7/STAT5/SOCS pathways. Nature.

[68] Chetoui N, Boisvert M, Gendron S, Aoudjit F (2010). Interleukin-7 promotes the survival of human CD4 + effector/memory T cells by up-regulating Bcl-2 proteins and activating the JAK/STAT signalling pathway. Immunology..

[69] Palmer DC, Restifo NP (2009). Suppressors of cytokine signaling (SOCS) in T cell differentiation, maturation, and function. Trends Immunol..

[70] Yoshimura A, Naka T, Kubo M (2007). SOCS proteins, cytokine signalling and immune regulation. Nat Rev Immunol..

[71] Wang L, Hui H, Agrawal K, Kang Y, Li N, Tang R, Yuan J, Rana TM (2020). m(6) A RNA methyltransferases METTL3/14 regulate immune responses to anti-PD-1 therapy. EMBO J..

[72] Le Poole IC, Riker AI, Quevedo ME, Stennett LS, Wang E, Marincola FM, Kast WM, Robinson JK, Nickoloff BJ (2002). Interferon-gamma reduces melanosomal antigen expression and recognition of melanoma cells by cytotoxic T cells. Am J Pathol..

[73] Ramesh P, Shivde R, Jaishankar D, Saleiro D, Le Poole IC (2021). A palette of cytokines to measure anti-tumor efficacy of T cell-based therapeutics. Cancers (Basel)..

[74] Jorgovanovic D, Song M, Wang L, Zhang Y (2020). Roles of IFN-gamma in tumor progression and regression: a review. Biomark Res.

[75] Farrar MA, Schreiber RD (1993). The molecular cell biology of interferon-gamma and its receptor. Annu Rev Immunol.

[76] Hiroaki Ikeda LJO, Robert D (2002). Schreiber The roles of IFN-y in protection against tumor development and cancer immunoediting. Cytokine Growth Factor Rev.

[77] Vinay DS, Ryan EP, Pawelec G, Talib WH, Stagg J, Elkord E, Lichtor T, Decker WK, Whelan RL, Kumara H (2015). Immune evasion in cancer: Mechanistic basis and therapeutic strategies. Semin Cancer Biol.

[78] Dunn GP, Bruce AT, Ikeda H, Old LJ, Schreiber RD (2002). Cancer immunoediting: from immunosurveillance to tumor escape. Nat Immunol.

[79] Bai R, Chen N, Li L, Du N, Bai L, Lv Z, Tian H, Cui J (2020). Mechanisms of Cancer Resistance to Immunotherapy. Front Oncol.

[80] Jhunjhunwala S, Hammer C, Delamarre L (2021). Antigen presentation in cancer: insights into tumour immunogenicity and immune evasion. Nat Rev Cancer.

[81] Burr ML, Sparbier CE, Chan KL, Chan YC, Kersbergen A, Lam EYN, Azidis-Yates E, Vassiliadis D, Bell CC, Gilan O (2019). An Evolutionarily Conserved Function of Polycomb Silences the MHC Class I Antigen Presentation Pathway and Enables Immune Evasion in Cancer. Cancer Cell..

[82] Dhatchinamoorthy K, Colbert JD, Rock KL (2021). Cancer immune evasion through loss of MHC Class I antigen presentation. Front Immunol..

[83] Yamamoto K, Venida A, Yano J, Biancur DE, Kakiuchi M, Gupta S, Sohn ASW, Mukhopadhyay S, Lin EY, Parker SJ (2020). Autophagy promotes immune evasion of pancreatic cancer by degrading MHC-I. Nature..

[84] Kroemer G, Zitvogel L (2020). Seeking Cellular Fitness and Immune Evasion: Autophagy in Pancreatic Carcinoma. Cancer Cell..

[85] Jin S, Zhang X, Miao Y, Liang P, Zhu K, She Y, Wu Y, Liu DA, Huang J, Ren J (2018). m(6)A RNA modification controls autophagy through upregulating ULK1 protein abundance. Cell Res..

[86] Wang X, Wu R, Liu Y, Zhao Y, Bi Z, Yao Y, Liu Q, Shi H, Wang F, Wang Y (2020). m(6)A mRNA methylation controls autophagy and adipogenesis by targeting Atg5 and Atg7. Autophagy.

[87] Lin Z, Niu Y, Wan A, Chen D, Liang H, Chen X, Sun L, Zhan S, Chen L, Cheng C (2020). RNA m(6) A methylation regulates sorafenib resistance in liver cancer through FOXO3-mediated autophagy. EMBO J..

[88] Zhou J, Ding T, Pan W, Zhu LY, Li L, Zheng L (2009). Increased intratumoral regulatory T cells are related to intratumoral macrophages and poor prognosis in hepatocellular carcinoma patients. Int J Cancer.

[89] Liu Z, Wang T, She Y, Wu K, Gu S, Li L, Dong C, Chen C, Zhou Y (2021). N6-methyladenosine-modified circIGF2BP3 inhibits CD8 + T-cell responses to facilitate tumor immune evasion by promoting the deubiquitination of PD-L1 in non-small cell lung cancer. Mol Cancer..

[90] Chinen T, Kannan AK, Levine AG, Fan X, Klein U, Zheng Y, Gasteiger G, Feng Y, Fontenot JD, Rudensky AY (2016). An essential role for the IL-2 receptor in Treg cell function. Nat Immunol..

[91] Lio CW, Hsieh CS (2008). A two-step process for thymic regulatory T cell development. Immunity..

[92] Tong J, Cao G, Zhang T, Sefik E, Amezcua Vesely MC, Broughton JP, Zhu S, Li H, Li B, Chen L (2018). m(6)A mRNA methylation sustains Treg suppressive functions. Cell Res..

[93] Zhou Y, Zhou H, Shi J, Guan A, Zhu Y, Hou Z, Li R (2021). Decreased m6A Modification of CD34/CD276(B7-H3) Leads to Immune Escape in Colon Cancer. Front Cell Dev Biol..

[94] Lei Y, Li X, Huang Q, Zheng X, Liu M (2021). Progress and Challenges of Predictive Biomarkers for Immune Checkpoint Blockade. Front Oncol.

[95] Wang Y, Zhang X, Wang Y, Zhao W, Li H, Zhang L, Li X, Zhang T, Zhang H, Huang H (2021). Application of immune checkpoint targets in the anti-tumor novel drugs and traditional Chinese medicine development. Acta Pharm Sin B..

[96] Li T, Ma R, Zhu JY, Wang FS, Huang L, Leng XS (2015). PD-1/PD-L1 Costimulatory Pathway-induced Mouse Islet Transplantation Immune Tolerance. Transpl P..

[97] Han YY, Liu DD, Li LH (2020). PD-1/PD-L1 pathway: current researches in cancer. Am J Cancer Res..

[98] Yang S, Wei J, Cui YH, Park G, Shah P, Deng Y, Aplin AE, Lu Z, Hwang S, He C (2019). m(6)A mRNA demethylase FTO regulates melanoma tumorigenicity and response to anti-PD-1 blockade. Nat Commun.

[99] Li N, Kang Y, Wang L, Huff S, Tang R, Hui H, Agrawal K, Gonzalez GM, Wang Y, Patel SP (2020). ALKBH5 regulates anti-PD-1 therapy response by modulating lactate and suppressive immune cell accumulation in tumor microenvironment. Proc Natl Acad Sci U S A..

[100] Su R, Dong L, Li Y, Gao M, Han L, Wunderlich M, Deng X, Li H, Huang Y, Gao L (2020). Targeting FTO Suppresses Cancer Stem Cell Maintenance and Immune Evasion. Cancer Cell..

[101] Klement JD, Paschall AV, Redd PS, Ibrahim ML, Lu C, Yang D, Celis E, Abrams SI, Ozato K, Liu K (2018). An osteopontin/CD44 immune checkpoint controls CD8 + T cell activation and tumor immune evasion. J Clin Invest..

[102] Melief CJM, Toes REM, Medema JP, Van der Burg SH, Ossendorp F, Offringa R (2000). Strategies for immunotherapy of cancer. Adv Immunol.

[103] Postow MA, Sidlow R, Hellmann MD (2018). Immune-Related Adverse Events Associated with Immune Checkpoint Blockade. N Engl J Med.

[104] Met O, Jensen KM, Chamberlain CA, Donia M, Svane IM (2019). Principles of adoptive T cell therapy in cancer. Semin Immunopathol.

[105] Vermaelen K (2019). Vaccine Strategies to Improve Anti-cancer Cellular Immune Responses. Front Immunol.

[106] Weber J (2007). Review: anti-CTLA-4 antibody ipilimumab: case studies of clinical response and immune-related adverse events. Oncologist..

[107] Topalian SL, Drake CG, Pardoll DM (2012). Targeting the PD-1/B7-H1(PD-L1) pathway to activate anti-tumor immunity. Curr Opin Immunol.

[108] He X, Xu C (2020). Immune checkpoint signaling and cancer immunotherapy. Cell Res..

[109] Han Y, Liu D, Li L (2020). PD-1/PD-L1 pathway: current researches in cancer. Am J Cancer Res..

[110] Villadolid J, Amin A (2015). Immune checkpoint inhibitors in clinical practice: update on management of immune-related toxicities. Transl Lung Cancer Res..

[111] Huang Y, Su R, Sheng Y, Dong L, Dong Z, Xu H, Ni T, Zhang ZS, Zhang T, Li C (2019). Small-Molecule Targeting of Oncogenic FTO Demethylase in Acute Myeloid Leukemia. Cancer Cell..

[112] Chen B, Ye F, Yu L, Jia G, Huang X, Zhang X, Peng S, Chen K, Wang M, Gong S (2012). Development of cell-active N6-methyladenosine RNA demethylase FTO inhibitor. J Am Chem Soc..

[113] Cui Q, Shi H, Ye P, Li L, Qu Q, Sun G, Sun G, Lu Z, Huang Y, Yang CG (2017). m(6)A RNA Methylation Regulates the Self-Renewal and Tumorigenesis of Glioblastoma Stem Cells. Cell Rep..

[114] Yankova E, Blackaby W, Albertella M, Rak J, De Braekeleer E, Tsagkogeorga G, Pilka ES, Aspris D, Leggate D, Hendrick AG (2021). Small-molecule inhibition of METTL3 as a strategy against myeloid leukaemia. Nature..

[115] Weng H, Huang H, Wu H, Qin X, Zhao BS, Dong L, Shi H, Skibbe J, Shen C, Hu C (2018). METTL14 Inhibits Hematopoietic Stem/Progenitor Differentiation and Promotes Leukemogenesis via mRNA m(6)A Modification. Cell Stem Cell..

[116] Wang Y, Lu JH, Wu QN, Jin Y, Wang DS, Chen YX, Liu J, Luo XJ, Meng Q, Pu HY (2019). LncRNA LINRIS stabilizes IGF2BP2 and promotes the aerobic glycolysis in colorectal cancer. Mol Cancer.

[117] Su R, Dong L, Li C, Nachtergaele S, Wunderlich M, Qing Y, Deng X, Wang Y, Weng X, Hu C (2018). R-2HG Exhibits Anti-tumor Activity by Targeting FTO/m(6)A/MYC/CEBPA Signaling. Cell..

[118] Qing Y, Dong L, Gao L, Li C, Li Y, Han L, Prince E, Tan B, Deng X, Wetzel C (2021). R-2-hydroxyglutarate attenuates aerobic glycolysis in leukemia by targeting the FTO/m(6)A/PFKP/LDHB axis. Mol Cell..

[119] Rowshanravan B, Halliday N, Sansom DM (2018). CTLA-4: a moving target in immunotherapy. Blood..

[120] Peggs KS, Quezada SA, Chambers CA, Korman AJ, Allison JP (2009). Blockade of CTLA-4 on both effector and regulatory T cell compartments contributes to the antitumor activity of anti-CTLA-4 antibodies. J Exp Med.

[121] Lipson EJ, Drake CG (2011). Ipilimumab: an anti-CTLA-4 antibody for metastatic melanoma. Clin Cancer Res..

[122] Ditsiou A, Cilibrasi C, Simigdala N, Papakyriakou A, Milton-Harris L, Vella V (2020). The structure-function relationship of oncogenic LMTK3. Sci Adv..

[123] Huang CT, Workman CJ, Flies D, Pan X, Marson AL, Zhou G, Hipkiss EL, Ravi S, Kowalski J, Levitsky HI (2004). Role of LAG-3 in regulatory T cells. Immunity.

[124] Ruffo E, Wu RC, Bruno TC, Workman CJ, Vignali DAA (2019). Lymphocyte-activation gene 3 (LAG3): The next immune checkpoint receptor. Semin Immunol.

[125] Meyers JH, Sabatos CA, Chakravarti S, Kuchroo VK (2005). The TIM gene family regulates autoimmune and allergic diseases. Trends Mol Med.

[126] Chauvin JM, Zarour HM (2020). TIGIT in cancer immunotherapy. J Immunother Cancer..

[127] Sharma P, Allison JP (2015). The future of immune checkpoint therapy. Science.

[128] Yan W, Hu H, Tang B (2019). Advances Of Chimeric Antigen Receptor T Cell Therapy In Ovarian Cancer. Onco Targets Ther.

[129] Alard E, Butnariu AB, Grillo M, Kirkham C, Zinovkin DA, Newnham L, Macciochi J, Pranjol MZI (2020). Advances in anti-cancer immunotherapy: Car-T cell, checkpoint inhibitors, dendritic cell vaccines, and oncolytic viruses, and emerging cellular and molecular targets. Cancers (Basel)..

[130] June CH, O’Connor RS, Kawalekar OU, Ghassemi S, Milone MC (2018). CAR T cell immunotherapy for human cancer. Science..

[131] Brentjens RJ, Davila ML, Riviere I, Park J, Wang X, Cowell LG, Bartido S, Stefanski J, Taylor C, Olszewska M (2013). CD19-targeted T cells rapidly induce molecular remissions in adults with chemotherapy-refractory acute lymphoblastic leukemia. Sci Transl Med.

[132] Grupp SA, Kalos M, Barrett D, Aplenc R, Porter DL, Rheingold SR, Teachey DT, Chew A, Hauck B, Wright JF (2013). Chimeric antigen receptor-modified T cells for acute lymphoid leukemia. N Engl J Med.

[133] Yilmaz A, Cui H, Caligiuri MA, Yu J (2020). Chimeric antigen receptor-engineered natural killer cells for cancer immunotherapy. J Hematol Oncol.

[134] Song H, Song J, Cheng M, Zheng M, Wang T, Tian S, Flavell RA, Zhu S, Li HB, Ding C (2021). METTL3-mediated m(6)A RNA methylation promotes the anti-tumour immunity of natural killer cells. Nat Commun..

[135] Gabrilovich DI, Nagaraj S (2009). Myeloid-derived suppressor cells as regulators of the immune system. Nat Rev Immunol.

[136] Li B, Jiang J, Assaraf YG, Xiao H, Chen ZS, Huang C (2020). Surmounting cancer drug resistance: New insights from the perspective of N(6)-methyladenosine RNA modification. Drug Resist Updat.

